# 16S rRNA Gene Sequence Analysis of V6–V8 Region Provides Limited Advantage in Diagnosis of Chronic Prostatitis

**DOI:** 10.3390/diagnostics15081003

**Published:** 2025-04-15

**Authors:** Jens Rosellen, Moritz Fritzenwanker, Hans-Christian Schuppe, Undraga Schagdarsurengin, Florian Wagenlehner, Adrian Pilatz

**Affiliations:** 1 Department of Urology, Pediatric Urology and Andrology, Justus Liebig University Giessen, 35392 Giessen, Germany; 2 Institute for Medical Microbiology, Justus Liebig University Giessen, 35390 Giessen, Germany

**Keywords:** chronic prostatitis, CPPS, 16S rRNA sequencing technique, microbiology

## Abstract

**Background**: 16S rRNA analysis has been used in various diseases to identify pathogenic bacteria. In particular, pathogens that are difficult to cultivate or previously unknown can be detected with great certainty. In chronic prostatitis/chronic pelvic pain syndrome (CP/CPPS), a distinction between bacterial and non-bacterial genesis is essential with regard to categorization and therapy. The objective of this study is to investigate the value of 16S rRNA gene sequence analysis in the routine management of patients with CP/CPPS especially after failure to detect a pathogen in conventional culture and polymerase chain reaction for sexually transmitted diseases (STI-PCR). **Methods**: In total, 228 patients with CP/CPPS were prospectively enrolled and received a comprehensive andrological work-up. Microbial analysis consisted of standard bacterial cultures and the detection of sexually transmitted pathogens by PCR using urine specimens from a 2-glass test and semen analysis. 16S rRNA gene sequence analysis was performed in patients with urine and semen of patients without bacterial pathogens in microbiological culture and STI-PCR. **Results**: In 184 of 199 (92%) patients with negative ejaculate culture and negative STI-PCR, no pathogen could be detected by 16S rRNA analysis and in the case of a positive result, the analysis only showed non-pathogenic bacteria of the normal flora. There was no statistical association between the 16S rRNA analysis and the inflammatory markers or the National Institutes of Health Chronic Prostatitis Symptom Index (NIH-CPSI) scores. **Conclusions**: At least in our study cohort, the 16S rRNA analysis provided no additional benefit following microbiological culture and STI-PCR in the categorization of patients with CP/CPPS.

## 1. Introduction

Chronic prostatitis is a frequent condition with lifetime prevalences ranging from 1.8 to 8.2% [1,2]. Risk factors include diseases that can cause neuropathic pain and disorders that make the patient more susceptible to urinary tract infections [3]. Patients with a history of urethritis brought on by sexually transmitted infections (STIs) and those with disorders that allow for retrograde ascension of bacteria into the urethra and prostate are considered to be at a higher risk of developing chronic prostatitis [3,4,5].

Prostatitis should be differentiated from other causes of pelvic pain, such as interstitial cystitis, benign prostate hyperplasia, and other causes of dysuria [6,7]. Acute bacterial prostatitis (category I), chronic bacterial prostatitis (category II), chronic nonbacterial prostatitis/chronic pelvic pain syndrome (CP/CPPS) (category III), and asymptomatic in-flammatory prostatitis (category IV) are the four categories into which the National Institutes of Health (NIH) has classified the condition [7]. Category III is further subdivided in type IIIA, which exhibits inflammatory characteristics in the ejaculate, and type IIIB, which does not [7]. More than 90% of cases with chronic prostatitis are categorized as CP/CPPS. Prostatic discomfort must be present for at least three months without any definitive microbiological findings [1,6,7,8,9].

An exact distinction between type II and type III is of clinical importance, since the former requires antibiotic therapy, whereas type III requires non-antibiotic, symptom-oriented treatment [7]. Diagnostic difficulties arise from the fact that the male urinary system is not completely sterile as it has been already shown that certain bacteria, such as *Staphylococcus epidermidis*, are present in otherwise healthy subjects [10,11]. Furthermore, even in healthy individuals, semen and urine samples may be contaminated by microorganisms of the normal flora during its passage through the genital tract, starting from the testes or bladder and expanding all the way to the meatus urethrae [11,12].

Against this background, reliable microbiological procedures are necessary in order to detect the pathogen causing an infection, even in the case of atypical pathogens, and at the same time to distinguish these from the normal physiological flora [8,12]. A clear distinction can prevent unnecessary antibiotic treatments [7]. The current standard in diagnostics is conventional microbiological cultures and STI-PCR, although false negative culture results after previous antibiotics can make precise diagnosis difficult and two different procedures are necessary [6,7,8,12].

For several decades, DNA sequencing of the bacterial 16S rRNA gene has been used to identify pathogenic and resident bacteria in various diseases and to assign phylogenetic relationships [13,14,15]. 16S rRNA gene sequence analysis can improve the identification of poorly described, rarely isolated, or phenotypically aberrant strains, can be routinely used for identification of mycobacteria, and can lead to the recognition of novel pathogens and non-culturable bacteria with high accuracy [13,16]. Despite its advantages, 16S rRNA gene sequence analysis lacks widespread use in clinical routine because of technical and cost considerations [16,17,18].

In studies, the method has been used to successfully detect pathogenic bacteria in cases of enteritis and respiratory infections [18,19,20]. Marshall et al. were able to demonstrate a high rate of concordance between the results of the 16S rRNA gene sequence analysis and the conventional urine culture in children with a suspected urinary tract infection [21]. Other studies showed better detection of pathogens in urinary tract infections compared to midstream urine culture [22,23]. The method was also successfully used to detect the spectrum of activity of antibiotics at the species level by measuring the number of bacteria in culture and was even able to differentiate between bactericidal and bacteriostatic effects of the antibiotics [24].

Due to its high accuracy, the 16S rRNA gene sequence analysis could complement or improve conventional microbiological cultures and prevent unnecessary antibiotic treatments [13,14]. Intracellular bacteria and biofilm formers, which are difficult to detect by conventional culture but are certainly relevant pathogens of prostatitis, could be reliably detected with this method [13,14,16]. Since the value of 16S rRNA analysis in CP/CPPS diagnostics is unclear, the aim of this study was to investigate this in a large prospective study. In particular, in the case of prior antibiotic pretreatment, where the conventional culture fails to detect any germs, as well as in the case of pathogens that are difficult to culture, the analysis could represent a useful supplement to conventional methods.

## 2. Materials and Methods

### 2.1. Study Population

This prospective study examined 312 patients who were referred to our tertiary university department between January 2020 and October 2024 for suspected chronic prostatitis as part of our special consultation for pelvic pain/chronic prostatitis. Beforehand, each included patient has given his written informed consent to participate in our study. A positive approval by the Institutional Ethics Committee of Justus-Liebig-University Giessen also has been received (protocol code 55/13, date of approval: 4 November 2013).

**Figure 1:**
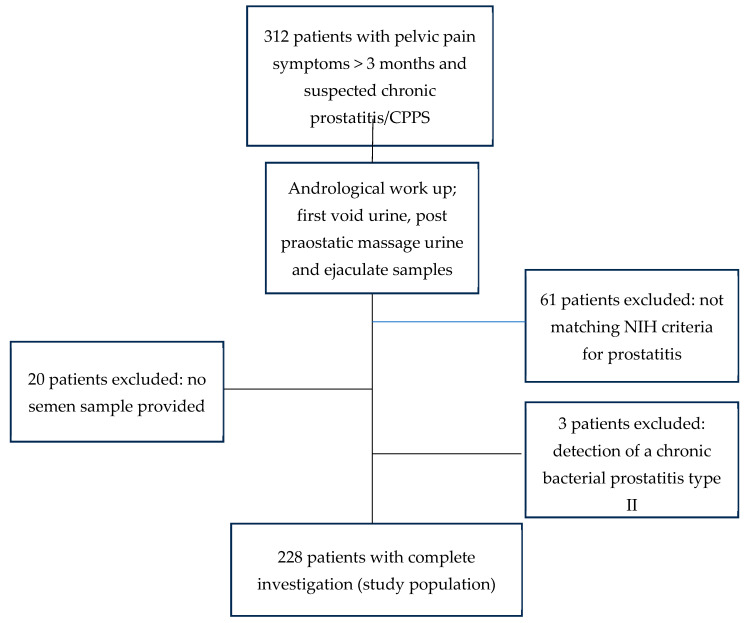
Composition of the study population.

Men who did not meet the diagnosis criteria for chronic prostatitis/CPPS (*n* = 61) and men with chronic prostatitis who were unable to provide a semen sample (*n* = 20) were not included in the study population. In three patients, *Chlamydia trachomatis* was detected in the STI-PCR, and appropriate antibiotic therapy was started. After successful eradication, all symptoms of pelvic pain ceased, so that they were accordingly classified as having chronic bacterial prostatitis type II and were also excluded from the study population. Thus, the study group consisted of 228 patients with CP/CPPS (Figure 1).

### 2.2. Clinical Investigations

As previously described each participant received a thorough andrological examination that included a structured review of their medical history, validated questionnaires for lower urinary tract symptoms (International Prostate Symptom Score, IPSS), erectile dysfunction (International Index of Erectile Function, IIEF), and chronic prostatitis (National Institutes of Health Chronic Prostatitis Symptom Index, NIH-CPSI), a physical examination, sex hormone analysis, a 2-glass urine test and additional semen analysis [25,26,27]. According to clinical recommendations, ultrasonography was used to assess the volumes of the prostate and testicles [28,29]. Anomalies were also meticulously documented.

### 2.3. Laboratory Methods

Every patient had routine blood draws to measure serum levels of prostate-specific antigen (PSA), estrogen, testosterone (normal range: 300–1000 ng/dL), C-reactive protein (CRP), and estradiol. The levels of prolactin, sex hormone-binding globulin (SHBG), albumin, follicle-stimulating hormone (FSH), luteinizing hormone (LH), and albumin were measured simultaneously using standard laboratory procedures in the central laboratory of the Giessen University Hospital (ADVIA and ADVIA Centaur, Siemens Health Care, Erlangen, Germany) if a lower testosterone level was discovered. Leukocyturia was detected using a urine dipstick and an automated quantitative urine particle analyzer (Cobas u 411, Roche Diagnostics GmbH, Basel, Switzerland). The technician who performed the assays was blind to the source of the material.

### 2.4. Routine Microbiological Tests and Semen Analysis

To exclude a bacterial pathogen as the cause of the pelvic pain, a conventional culture and a PCR for sexually transmitted pathogens were performed from first void urine, post-prostatic massage urine and ejaculate. In the case of a negative culture and a negative STI-PCR, a 16S rRNA gene sequence analysis was also performed. A bacterial count of over 1000 colony-forming units (CFU) per milliliter of ejaculate was considered relevant for bacteriospermia [26]. For the urine samples a germ count of 100 colony-forming units per ml was deemed significant [30].

Within an hour of collection, a blind analysis of the semen was conducted in accordance with WHO 2010 and WHO 2021 guidelines, with methodologies for basic semen parameters remaining unchanged [31,32]. Following patient instruction and glans and foreskin disinfection, the samples were collected at the clinic by masturbating into a sterile container. As previously described, all patients had their urine (first void urine, urine after prostatic massage) and semen tested for sexually transmitted infections (STIs) (*Mycoplasma genitalium*, *Mycoplasma hominis*, *Ureaplasma urealyticum*, *Ureaplasma parvum*, *Chlamydia trachomatis*, *Neisseria gonorrhoeae*, and *Trichomonas vaginalis*) and given bacterial cultures in order to rule out the presence of infections [25]. As mentioned, bacteriospermia was defined as having more than 1000 colony-forming units per milliliter of ejaculate according to WHO [31,32]. As part of standard processing, the concentration of leukocytes that tested positive for peroxidase was determined (Leucoscreen, FertiPro).

Additionally, polymorphonuclear (PMN) elastase, a marker of local inflammation, was measured in cell-free seminal plasma using an enzyme-linked immunoassay in each semen sample (Demeditec Diagnostics GmbH, Kiel, Germany). Measurement of inflammatory cytokine interleukin-8 (IL-8) concentrations was performed using the cytometric bead array (CBA) (BD Biosciences, San Jose, CA, USA). As described earlier [33], spectrophotometric techniques were used to measure the amounts of neutral α-glucosidase and fructose (total enzymatic activity).

### 2.5. DNA Extraction and 16S rRNA Gene Sequence Analysis

16S rRNA gene sequence analysis on first void urine, urine after prostatic massage, and ejaculate was performed on all cases in absence of a positive microbiological culture and STI-PCR [34,35].

We extracted DNA from urine and semen samples of prostatitis patients using the automated DNA/RNA-isolation system eMAG (Biomerieux, Marcy-l’Étoile, France). A total of 500 µL urine sample is mixed with 500 µL lysis buffer (Nuclisens easyMAG lysis buffer, Bioerieux, France). DNA is isolated using a customized program on the eMAG.

We then tested for bacterial DNA with a PCR targeting a region in the 16S-rRNA-Gene with the primers 0933-F: (5′-GCA CAA GCG GTG GAG CAT GTG G-3′) and 1407-R (5′-GAC GGG CGG TGT GTA CAA G-3′) [30].

PCR was performed with 10 µL Phusion-Mix (Thermo Scientific Phusion Flash High-Fidelity PCR Master Mix, Thermo Fischer, Waltham, Massachusetts, USA), 6 µL aqua dest, and 1 µL of each primer (10 pmol), plus 2 µL of the sample. The PCR was run on a ProFlex PCR System (appliedbiosystems, Thermo Fisher Scientific) with the following program: 98 °C 10 min, 30 cycles of 98 °C for 1 s, −68 °C for 5 s, −72 °C for 10 s, then 72 °C for 1 min. We checked for PCR products with laboratory standard agarose gel electrophoresis. PCR amplicons of PCR-positive samples were cleaned up with the MSB Spin PCRapace/Invisorb Fragment CleanUp kit (Stratec) and sent for Sanger sequencing to Microsynth company. For the sequencing reaction we used 0942-F (5′-CGG TGG AGC ATG TGG TTT AAT TCG-3′; primer designed and recommended by the sequencing company to improve the sequencing reaction) and the aforementioned reverse primer. Forward and reverse reads were checked for quality. Electropherograms were checked for inconsistencies that would signal a mixture of sequences, potentially caused by the presence of multiple species in the sample; such sequences were excluded from further analysis. Forward and reverse reads were assembled into a consensus sequence using CLC Genomics Workbench Sequence Viewer 4.0. The consensus sequences were then manually analyzed online at the NCBI website using nucleotide megablast on the nucleotide collection (National Center for Biotechnology Information (NCBI), Bethesda, MD, USA, available online: https://www.ncbi.nlm.nih.gov/, accessed on 12 February 2025). Results were inspected for percent identity, query coverage, and E-value. Additionally, consensus sequences were analyzed at the Sepsitest website (https://sepsitest-blast.com/de/index.php, Molzym, Germany, accessed on 12 February 2025).

### 2.6. Statistical Analysis

The statistical analysis was conducted using SPSS 29 for Windows (IBM GmbH, Ehningen, Germany).

Since data were not normally distributed, the Mann–Whitney U test and the Kruskal–Wallis test were applied.

The Mann–Whitney U test was used to compare patients with chronic prostatitis without any microbiological findings and patients with at least one positive result in first void urine, post-prostatic massage urine, or ejaculate. A value of *p* < 0.05 was considered statistically significant.

The correlation between the previously mentioned parameters and the microbiological subgroups of the ejaculate samples was tested using the Kruskal–Wallis test. A value of *p* < 0.05 was considered statistically significant.

Multivariate regression modeling was used to examine the association between the results of a positive microbiological culture, STI-PCR and 16S rRNA analysis of the ejaculate samples and various seminal inflammatory parameters, sperm concentration, and the CPSI total score. Only non-missing data were included in the modeling exercise using a forward stepwise process. A value of *p* < 0.05 was considered statistically significant.

## 3. Results

### 3.1. Demographics

Table 1 displays the comprehensive clinical and demographic results. The patients’ ages ranged from 20 to 66 years old, with a median age of 37. The majority of patients (88.2%) had chronic prostatitis type IIIB, whereas 11.8% of the study group had type IIIA chronic prostatitis.

### 3.2. Questionnaires

The study group had a medium degree of lower urinary tract symptoms, as shown by the median score of 10 points on the International Prostate Symptom Score (IPSS). The International Index of Erectile Function (IIEF) had a median score of 28 points, which was within the normal range. The National Health Institute’s Chronic Prostatitis Symptom Index (NIH-CPSI) showed a medium symptom burden from chronic prostatitis, with median scores of 12 points for pain (CPSI-I), 3 points for urinary tract symptoms (CPSI-II), and 9 points for impact on quality of life (CPSI-III). However, not all patients were able to complete the surveys because of language barriers, and patients who did not engage in sexual activity were also unable to complete the IIEF-5 questionnaire in a meaningful way.

### 3.3. Andrological Results

The average testicular volume was 15.0 mL and within the normal range [28]. The same is true for the median prostate volume, which was 20.0 mL [29].

With median values of total testosterone at 460 ng/dL, PSA at 0.67 ng/mL, estradiol at 31 pg/mL, and c-reactive protein (CRP) at 0.5 mg/L, the laboratory parameters were within normal limits.

The WHO lower reference limits for the basic semen variables are shown in Table 2, along with the patients’ semen parameters. There were no indications of inflammatory processes in the study population, as evidenced by the median values of all the cohort’s evaluated semen parameters falling within the normal range, particularly the seminal markers for inflammation interleukin-8, elastase, and peroxidase-positive leukocytes. However, because the ejaculate volume was occasionally too low, not all parameters could always be determined in all cases.

### 3.4. Microbiology

**Figure 2:**
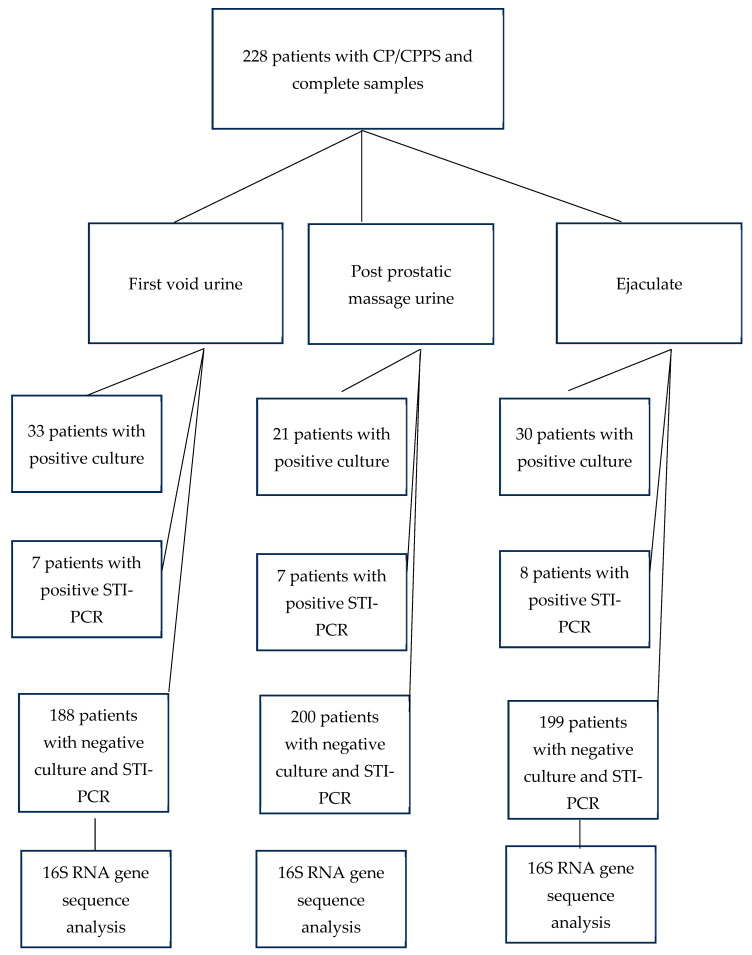
Patient distribution according to the results of the microbiological investigations of the samples.

For each sample type, three groups were defined: those with positive microbiological culture, those with positive STI-PCR, and a third group in which culture and PCR did not detect pathogens and who also received a 16S RNA gene sequence analysis. An overview of patient distribution is provided in Figure 2.

There were only *n* = 8 patients with a positive STI in our study population. In contrast to the three patients with chronic prostatitis type II, in whom antibiotic treatment led to a disappearance of the pelvic pain symptoms and which were excluded from the study population as shown in Figure 1, antimicrobial therapy did not lead to a clinical improvement of the symptoms in these patients. Even after successful pathogen eradication, symptoms typical of CPPS were still reported in the follow-up after 6 to 12 months.

Appendix A Table A1 shows the pathogens detected in the first void urine broken down by microbiological culture, STI-PCR and 16S rRNA analysis. Due to our study cohort that had been extensively pretreated with antibiotics, the conventional culture was only positive in 33 patients. The pathogens detected were mostly contaminations or part of the normal flora, with a mostly low bacterial count of between 100 and 1000 CFU per milliliter. Likewise, the STI-PCR was only positive in 7 patients, with only *Ureaplasma urealyticum* being detected. The 16S rRNA analysis carried out in the case of a negative culture and STI-PCR also failed to detect any pathogens in 90% of cases; in the case of a positive detection (10% of cases), germs from the normal flora were detected.

Similarly, Appendix A Table A2 shows the microbiological results of the post-prostatic massage urine. In the case of a positive result in culture (*n* = 21) and 16S rRNA analysis (*n* = 14), urethral mucosal flora was detected in most cases. Moreover, *Urealplasma ureatyticum* was detected in a small proportion of the subjects in the STI-PCR (*n* = 7), similar to results obtained from first void urine samples. The vast majority (186 of 228 patients, 82%) of the analyses showed no detectable pathogen.

The results for the ejaculate are shown in Appendix A Table A3. Again, only very few pathogens were identified by culture (*n* = 30), STI-PCR (*n* = 8), and 16S rRNA analysis (*n* = 15).

Appendix A Table A4 shows a univariate and multivariate analysis between positive ejaculate culture and sperm concentration as an important fertility marker and the parameters of seminal inflammation and the CPSI total score. There was no significant association with any of the parameters examined, except for leukocytes in the univariate analysis. A *p* < 0.05 was considered statistically significant.

Appendix A Table A5 and Table A6 performed the same analysis with positive ejaculate STI-PCR and positive ejaculate 16S rRNA analysis. Again, no association with the parameters collected was found.

In order to investigate the impact of the detection of bacteria in the ejaculate on sperm concentration, inflammatory seminal parameters, and CPSI total score, patients with positive and negative microbiological analyses were compared. According to the World Health Organization [31,32], a bacterial count exceeding 1000 CFU per milliliter of ejaculate was considered pertinent for bacteriospermia. Due to the previous treatment of our patients by the referring physician, only eight patients of our study population had a positive STI polymerase chain reaction (PCR) test result. The majority of them (seven out of eight patients) were positive for U. urealyticum. The control group included all cases without a bacterial pathogen in culture, a negative STI-PCR, and a negative 16S rRNA analysis from semen. The other groups consisted of patients with positive STI-PCR, positive 16S rRNA analysis, and patients with bacteriospermia/positive ejaculate culture.

Table 3 demonstrates the association between abnormal microbiological findings in the analyses and inflammatory semen parameters as well as sperm concentration and CPSI total score along with the WHO 2021 lower reference limits. Neither a positive semen culture nor a positive STI-PCR or 16S analysis showed an association with the compared parameters. Again a *p* < 0.05 was deemed statistically significant.

In order to further investigate the influence of a positive microbiological finding on the study population, the study cohort was divided into two subgroups: those patients who never showed any evidence of bacteria in any of the three samples (first urine, massage urine, ejaculate) by any of the three microbiological methods (conventional culture, STI-PCR, 16S analysis) and those in with at least one positive bacterial finding in any sample category.

Table 4 shows the results for the demographic and andrological parameters in patients with no microbiological findings and those with at least one positive result. All laboratory parameters are within the normal reference values in both groups, as are the volumes of the testes and prostate. In both groups there are mild to moderate urination problems with slightly impaired erectile function and significant pelvic pain symptoms based on the scores of the questionnaires. The Mann–Whitney U test was used to compare the differences between the groups, and *p* < 0.05 was deemed statistically significant. As demonstrated, there are no significant differences between the two groups.

The outcomes of the two groups’ semen analyses are shown in Table 5 along with the WHO 2021 lower reference values. The assessed semen parameters, especially the inflammatory markers elastase, peroxidase-positive leukocytes, and interleukin-8, all had median values within the normal range. This suggests that the study population did not exhibit any signs of inflammatory processes. However, because the ejaculate volume was occasionally too low in some samples, not all parameters could be determined in all patients. Again, the Mann–Whitney U test was used for comparing the two groups, and *p* < 0.05 was deemed statistically significant.

## 4. Discussion

As a continuation of our previous publications [26], our study is the first to systematically investigate the role of 16S rRNA analysis in the diagnostic algorithm in patients suffering from chronic prostatitis/CPPS who were sent to our department for diagnosis and further treatment. In recent studies, the 16S rRNA analysis showed similarly reliable results compared to conventional microbiological culture in detecting the causative pathogen in bacterial enteritis and pneumonia [18,19,20]. In childhood urinary tract infections, the analysis also showed at least equivalent detection of bacteria compared to urine culture, with clear advantages in addition to the speed of diagnosis and the successful detection of small numbers of bacteria [22,23,36]. Goel et al. were able to demonstrate reliable bacteria detection from as few as 100 coliform units per milliliter, so that bacterial identification was possible even in early infections or after antibiotic treatment had already begun [36].

However, in our study population, we were unable to demonstrate any benefit of the analysis as an additional investigation in the case of a negative conventional culture and negative STI-PCR. In the case of first-void urine, post-prostatic massage urine, and ejaculate, the analysis failed to detect any bacteria in over 90% of cases. In the case of a positive bacterial detection, almost exclusively non-pathogenic bacteria of the normal urogenital flora were detected, so that no treatment consequence resulted [37]. Even the rare detection of potentially pathogenic bacteria such as *E. coli* or enterococci was considered by us to be contamination due to the low number of bacteria not detected via culture. Although not ultimately conclusive, the frequent pretreatment by an external colleague with antibiotics without any improvement in symptoms supports this conclusion. In line with this, a positive 16S rRNA analysis of the ejaculate samples showed no association with inflammatory semen parameters such as peroxidase-positive leukocytes, elastase, or IL-8, which also suggests contamination. Sperm concentration as an important parameter of ejaculate quality as well as the symptom burden of chronic pelvic pain, measured by the CPSI score, also showed no association. One reason for this could be the massive antibiotic pretreatment of our study population. Most of our subjects had previously received antibiotic treatment for CP/CPPS, although they had to be free of antibiotics for at least two weeks at the time of presentation to our consultation. Nevertheless, some of the germs we detected could have potentially pathogenic effects, so that follow-up studies are necessary to further characterize the urethral flora and its possible involvement in the development and maintenance of chronic prostatitis. There were also only a few subjects in the cohort with positive cultures in the first void urine (*n* = 33), post prostatic massage urine (*n* = 21), and ejaculate, (*n* = 30) so that due to the small numbers, no statistical associations could be seen. This applies in particular to the results of the STI-PCR, where only seven patients showed almost exclusively *Ureaplasma urealyticum*. Another factor could be the 16S rRNA analysis method used itself, which may have been too insensitive to reliably detect low numbers of bacteria. Likewise, in our study cohort, non-bacterial causes may be more prevalent in the development and maintenance of chronic pelvic pain syndrome, which could also explain the low level of bacterial detection.

Our study also did not directly compare the 16S rRNA analysis with conventional culture and STI-PCR; we only used it as a supplement to these methods in the case of previous negative results. Based on our study design, neither superiority nor inferiority compared to culture and STI-PCR can be derived for the analysis in the context of the primary diagnosis of a bacterial pathogen. A future study could compare the detection of bacteria directly between 16S rRNA analysis on the one hand and culture and STI-PCR on the other.

Strikingly, despite a positive conventional ejaculate culture or a positive ejaculate STI-PCR, no reduction in the sperm concentration or an elevation of the inflammatory seminal parameters could be detected. In contrast to our observation, various culture-based studies have been able to demonstrate a negative influence of bacteriospermia and STIs on ejaculate quality [38,39,40]. One explanation for this would again be the low number of patients with positive germ detection. In one third of the cases, only mixed flora and components of the normal flora such as streptococci were found in the ejaculate culture [10,11,12], so that contaminations rather than relevant infections were detected given the generally low number of germs. In the case of STI-PCR, *U. urealyticum* was almost exclusively identified as the pathogen. Older studies found only a slight negative influence of this pathogen on the ejaculate parameters, while inflammatory parameters were unremarkable [41,42]. This is consistent with our observations.

A clear limitation of our study is the single-center data collection and the lack of systematic recording of prior antibiotic treatment. Furthermore, we did not examine the entire 16S rRNA gene or multiple regions but focused only on the 0933-F and 1407-R regions.

For clinical practice, an additional 16S rRNA analysis after negative conventional culture and STI-PCR did not provide any additional insight in our study cohort. Nevertheless, the method can be helpful as a replacement for culture and PCR, if a pathogen that is difficult to culture is suspected, or in the case of ongoing antibiotic treatment, where cultural pathogen detection is often not possible.

Future work could, therefore, focus on the 16S rRNA analysis as a replacement for culture and STI-PCR or its use in patients with recurrent or persistent urogenital infection. Also, the analysis of the microbiome of CP/CPPS patients could also be carried out using this method or by using a more comprehensive methods like metagenomic sequencing.

## 5. Conclusions

The results of the large prospective study show that additional 16S rRNA analysis is not helpful in patients with CP/CPPS because it does not provide any clinical benefit.

## Figures and Tables

**Table 1:** Demographic and andrological findings of the study population.

Parameter	Median (IQR) or *n* (%)	Number of Patients
Age (years)	37 (30–46)	228
Type of prostatitis		228
Type IIIA	11.8%	
Type IIIB	88.2%	
IPSS (points)	10 (6–16)	201
IIEF (points)	28 (22–30)	142
CPSI-I (points)	12 (8–14)	220
CPSI-II (points)	3 (2–6)	220
CPSI-III (points)	9 (7–11)	220
CPSI total score (points)	24 (19–28)	220
Total testosterone (ng/dL)	460 (352–563)	226
PSA (ng/mL)	0.67 (0.45–1.0)	224
Estradiol (pg/mL)	31 (26–27)	225
CRP (mg/L)	0.5 (0.5–1.8)	225
Testicular volume (mL)	15.0 (13–18)	227
Prostate volume (mL)	20.0 (16–25)	225

IQR: interquartile range.

**Table 2:** Semen parameters of the study population compared with WHO 2021 reference values [32].

Parameter	Patients with Chronic Prostatitis/CPPS (*n* = 228)	WHO 2021 Reference Values	Number of Patients
Volume	2.5 (1.5–3.8)	1.4 ^1^	228
pH value	7.8 (7.5–8.0)	≥7.2 ^2^	228
Sperm concentration (10^6^/mL)	52.5 (20.5–115.9)	16 ^1^	228
Total sperm count (10^6^/ejaculate)	127.5 (34.7–278.7)	39 ^1^	228
Progressive motility (%)	49 (35–56)	30 ^1^	179
Sperm vitality (%)	60 (54–75)	58 ^1^	61
Normal forms (%)	12 (7–16)	4 ^1^	211
α-glucosidase (mU/ejaculate)	46.9 (25.1–76.5)	≥20/ejaculate ^2^	225
Fructose (µmol/ejaculate)	26.8 (9.7–49.8)	≥13/ejaculate ^2^	225
	7.0 (3.8–13.8)	≥2.4/ejaculate ^2^	225
Peroxidase-positive leukocytes (10^6^/mL)	0.2 (0–0.5)	<1 ^2^	228
Elastase (ng/mL)	43.0 (14–121)	<250 ^3^	223
Interleukin-8 (pg/mL)	3308.0 (2061–5804)	<10,000 ^3^	223

^1^ Lower reference limit based on 5th percentile. ^2^ Consensus-based reference values. ^3^ Threshold levels established in the Giessen Andrology laboratory.

**Table 3:** Association of microbiological findings in ejaculate with CPSI total score, sperm concentration and inflammatory seminal parameters.

Parameter	Patients with Negative 16S rRNA Analysis (*n* = 184)	Patients with Positive Microbiology in Semen (*n* = 30)	Patients with Positive PCR for STI (*n* = 8)	Patients with Positive 16S rRNA (*n* = 15)	WHO 2021 Reference Values	*p* ^1^
CPSI total score	23.8 (19–28)	23.1 (19–28)	23.1 (14–28)	23.2 (14–28)	n.a.	0.537
Sperm concentration (10^6^/mL)	88.4 (20.8–126)	60.0 (8.9–926)	93.6 (18.8–157.5)	113.1 (48.6–144.3)	16 ^2^	0.095
Peroxidase-positive leukocytes (10^6^/mL)	0 (0–1)	1 (0–2)	0 (0–1)	0 (0–0)	0 ^3^	0.197
Elastase (ng/mL)	42 (14–119)	40 (15–206)	59 (18–157)	37 (10–77)	<250 ^4^	0.789
Interleukin-8 (pg/mL)	4893 (2085–5854)	5599 (1986–7379)	5456 (2820–6312)	4609 (2143–7428)	<10,000 ^4^	0.505

^1^ Kruskal–Wallis test comparing all four defined groups. ^2^ Based on lower 5th percentiles. ^3^ Consensus parameters. ^4^ Local lab criteria. n.a., not applicable.

**Table 4:** Comparison of demographic and andrological findings in patients with chronic prostatitis without any microbiological findings and patients with at least one positive result in first void urine, post-prostatic massage urine, or ejaculate.

Parameter	Patients with No Microbiological Findings (*n* = 134)	Patients with at Least One Positive Finding (*n* = 94)	*p* ^1^
Age (years)	39 (31–47)	37 (30–44)	0.549
IPSS (points)	11 (6–16)	12 (6–17)	0.822
IIEF (points)	25 (22–30)	24 (21–30)	0.959
CPSI (points)	24 (19–29)	25 (19–29)	0.619
Total testosterone (ng/mL)	446 (330–539)	496 (385–604)	0.231
PSA (ng/mL)	0.91 (0.46–1.08)	0.73 (0.44–0.93)	0.439
Estradiol (pg/mL)	31 (26–36)	33 (26–37)	0.453
CRP (mg/L)	1.44 (0.5–1.52)	2.1 (0.5–2.2)	0.501
Mean testicular volume (mL)	15.3 (12–18)	15.2 (13–18)	0.401
Prostate volume (mL)	21.4 (17–26)	18.6 (16–25)	0.212

^1^ Mann–Whitney U test comparing patients with chronic prostatitis without any microbiological findings and patients with at least one positive result in first void urine, post-prostatic massage, urine, or ejaculate.

**Table 5:** Comparison of Semen parameters in patients with chronic prostatitis without any microbiological findings and patients with at least one positive result in first void urine, post-prostatic massage urine, or ejaculate compared with WHO 2021 reference values.

Parameter	Patients with No Microbiological Findings (*n* = 134)	Patients with at Least One Positive Finding (*n* = 94)	WHO 2021 Reference Values	*p* ^1^
Volume (mL)	2.8 (1.5–4.0)	2.7 (1.3–3.5)	1.4 ^2^	0.781
pH value	7.8 (7.5–8.0)	7.9 (7.5–8.4)	≥7.2 ^3^	0.348
Sperm concentration (10^6^/mL)	52.5 (21.0–133.8)	53.7 (13.4–110.3)	16 ^2^	0.667
Total sperm count (10^6^/ejaculate)	132.8 (38.4–295.2)	120.2 (29.9–279.8)	39 ^2^	0.594
Progressive motility (%)	44 (39–57)	43 (32–57)	30 ^2^	0.937
Sperm vitality (%)	65 (53–77)	61 (54–73)	58 ^2^	0.465
Normal forms (%)	11 (7–12)	12 (7–16)	4 ^2^	0.755
α-glucosidase (mU/ejaculate)	48.1 (27.7–79.8)	54.8 (21.8–73.1)	≥20/ejaculate ^3^	0.525
Fructose (µmol/ejaculate)	28.6 (10.3–50.8)	35.2 (8.8–45.7)	≥13/ejaculate ^3^	0.459
Peroxidase-positive leukocytes (10^6^/mL)	0.5 (0–1)	0.5 (0–1)	<1 ^3^	0.265
Elastase (ng/mL)	45.0 (12.3–132.8)	43.1 (17.0–151.1)	<250 ^4^	0.988
Interleukin-8 (pg/mL)	4814 (2146–5813)	5374 (2068–6830)	<10,000 ^4^	0.326

^1^ Mann–Whitney U test comparing patients with no microbiological findings and at least one positive finding. ^2^ Lower reference limit based on 5th percentile. ^3^ Consensus-based reference values. ^4^ Threshold levels established in the Giessen Andrology laboratory.

## Data Availability

The data that support the findings of this study are available from the corresponding author upon reasonable request.

## Appendix A

**Table A1:** Microbiological findings in patients with positive first void urine culture, positive STI-PCR, and patients with negative culture and negative STI-PCR who received 16S rRNA detection.

Pathogen	Patients with Positive Microbiology in First Void Urine, *n* (%) (*n* = 33)	Patients with Positive STI-PCR, *n* (%) (*n* = 7)	Patients WHO Received a 16S rRNA Analysis, *n* (%) (*n* = 188)
Mixed flora ^1^	14 (42)		
*Streptococcus mitis*	11 (33)		1 (0.5)
*Escherichia coli*	3 (9)		
*Enterococcus faecalis*	3 (9)		
*Klebsiella pneuminae*	2 (6)		
*Urealplasma urealyticum*		7 (100)	
No pathogen detected			169 (90)
*Fusobacterium nucleatum*			12 (6)
*Baterioidaceae* species			5 (3)
*Finegoldia magna*			1 (0.5)

^1^ Detection of three or more bacterial species.

**Table A2:** Microbiological findings in patients with positive post prostatic massage urine culture, positive STI-PCR, and patients with negative culture and negative STI-PCR who received 16S rRNA detection.

Pathogen	Patients with Positive Microbiology in Post Prostatic Massage Urine, *n* (%) (*n* = 21)	Patients with Positive STI-PCR, *n* (%) (*n* = 7)	Patients Who Received a 16S rRNA Analysis, *n* (%) (*n* = 200)
Mixed flora	6 (29)		
*Streptococcus mitis*	4 (19)		5 (3)
*Staphyloccocus epidermidis*	4 (19)		
*Escherichia coli*	2 (10)		
*Enterococcus faecalis*	2 (9)		
*Klebsiella pneumoniae*	2 (9)		
*Morganella morganii*	1 (5)		
*Ureaplasma urealyticum*		7 (100)	
No pathogen detected			186 (93)
*Baterioidaceae* species			8 (4)
*Gardnerella vaginalis*			1 (0.5)

**Table A3:** Microbiological findings in patients with positive ejaculate culture, positive STI-PCR, and patients with negative culture and negative STI-PCR who received 16S rRNA detection.

Pathogen	Patients with Positive Microbiology in Ejaculate, *n* (%) (*n* = 30)	Patients with Positive STI-PCR, *n* (%) (*n* = 8)	Patients Who Received a 16S rRNA Analysis, *n* (%) (*n* = 199)
Mixed flora	10 (33)		
*Streptococcus mitis*	6 (20)		1 (0.5)
*Enterococcus faecalis*	6 (20)		1 (0.5)
*Staphylococcus epidermidis*	4 (13)		1 (0.5)
*Escherichia coli*	2 (7)		1 (0.5)
*Klebsiella* species	2 (7)		
*Ureaplasma urealyticum*		7 (88)	
*Mycoplasma hominis*		1 (12)	
No pathogen detected			184 (92)
*Lactobacillus iners*			8 (4)
*Fusobacterium nucleatum*			2 (1)
*Corynebacterium striatum*			1 (0.5)
*Finegoldia magna*			1 (0.5)

**Table A4:** Association of ejaculate culture with CPSI total score, sperm concentration, and inflammatory seminal parameters.

	Correlation Coefficient r	*p* (Univariate)	Correlation Coefficient β	*p* (Multivariate)
CPSI total score	−0.038	0.575	−0.029	0.668
Sperm concentration	−0.091	0.169	−0.103	0.132
Leukocytes in ejaculate	0.218	0.001	−0.017	0.806
Seminal plasma elastase	0.012	0.856	−0.055	0.444
Seminal plasma IL-8	0.04	0.555	0.066	0.359

Multivariate analysis: univariate and multivariate regression analysis between positive ejaculate culture and seminal parameters and CPSI score, *n* = 30.

**Table A5:** Association of ejaculate STI-PCR with CPSI total score, sperm concentration, and inflammatory seminal parameters.

	Correlation Coefficient r	*p* (Univariate)	Correlation Coefficient β	*p* (Multivariate)
CPSI total score	−0.013	0.845	−0.151	0.880
Sperm concentration	−0.029	0.665	0.150	0.881
Leukocytes in ejaculate	−0.017	0.806	0.014	0.989
Seminal plasma elastase	0.006	0.928	−0.055	0.446
Seminal plasma IL-8	0.043	0.519	0.037	0.609

Multivariate analysis: univariate and multivariate regression analysis between ejaculate STI-PCR and seminal parameters and CPSI score, *n* = 8.

**Table A6:** Association of positive ejaculate 16S rRNA analysis with CPSI total score, sperm concentration, and inflammatory seminal parameters.

	Correlation Coefficient r	*p* (Univariate)	Correlation Coefficient β	*p* (Multivariate)
CPSI total score	−0.03	0.672	−0.052	0.462
Sperm concentration	0.086	0.209	0.014	0.843
Leukocytes in ejaculate	−0.036	0.602	−0.056	0.430
Seminal plasma elastase	−0.041	0.555	−0.218	0.827
Seminal plasma IL-8	0.027	0.696	−0.021	0.776

Multivariate analysis: univariate and multivariate regression analysis between ejaculate 16S rRNA analysis and seminal parameters and CPSI score, *n* = 15.
